# Increasing Adiponergic System Activity as a Potential Treatment for Depressive Disorders

**DOI:** 10.1007/s12035-019-01644-3

**Published:** 2019-05-28

**Authors:** Douglas Affonso Formolo, Thomas Ho-Yin Lee, Suk-Yu Yau

**Affiliations:** 1 Department of Rehabilitation Sciences, Faculty of Health and Social Sciences, The Hong Kong Polytechnic University, 11 Yuk Choi Road, Hung Hom, Kowloon Hong Kong S.A.R.; 2 University Research Facility in Behavioral and Systems Neurosciences, The Hong Kong Polytechnic University, 11 Yuk Choi Road, Hung Hom, Kowloon Hong Kong S.A.R.

**Keywords:** Adiponectin, Adiponectin receptors, AdipoRon, Hippocampus, Depression, Neuroplasticity

## Abstract

Depression is the most devastating mental disorder and one of the leading contributors to the global medical burden. Current antidepressant prescriptions present drawbacks, including treatment resistance, delayed onset of treatment response, and side effects. The rapid and long-lasting antidepressant effect of ketamine has brought hope to treatment-resistant major depressive disorder patients. However, ketamine has undesirable addictive properties and is a drug of abuse. There is an urgent need, therefore, to develop novel pharmacological interventions that could be as effective as ketamine, but without its side effects. Adiponectin, a pleiotropic adipocyte-secreted hormone, has insulin-sensitizing and neurotrophic properties. It can cross the blood-brain barrier and target multiple brain regions where the adiponectin receptors are detected. Emerging evidence has suggested that adiponectin and the adiponectin receptor agonist, AdipoRon, could promote adult neurogenesis, dendritic and spine remodeling, and synaptic plasticity in the hippocampus, resulting in antidepressant effects in adult mice. By summarizing the most recent clinical and animal studies, this review provides a timely insight on how modulating the adiponergic system in the hippocampus could be a potential therapeutic target for an effective and fast-acting antidepressant response.

**Electronic supplementary material:**

The online version of this article (10.1007/s12035-019-01644-3) contains supplementary material, which is available to authorized users.

Depression is the worldwide, leading cause of years lived with disability, accounting for almost 10% of the total disability in 2010 [1, 2]. This devastating disorder imposes a huge economic burden on patients and the healthcare systems [3, 4]. Therapeutic effects of the currently available antidepressant treatments are limited considering their low response rates (~ 50%) and delayed therapeutic effects [5, 6]. The delayed antidepressant response is alarming due to an increased risk for suicidal behavior during the first month of antidepressant treatment [7]. Also, about 20% of the depressed patients remain treatment-resistant, failing to respond to at least four drug trials and two drug classes [4]. Among those, suicidal ideation increases from 6 to 15% and the average response rate is lowered to 36% [4]. With the unmet need for antidepressant treatments, there is an urge to develop novel antidepressant drugs.

Currently, depression is considered as a neurocognitive disorder with associated impairments in adult neurogenesis and neural circuits [8–10]. This emerging conceptualization of depression has guided the development of a novel class of antidepressants targeting structural and functional neuroplasticity. Ketamine, an *N*-methyl-d-aspartate (NMDA)−receptor antagonist [11], induces a rapid and long-lasting antidepressant response in treatment-resistant patients with major depressive disorder (MDD) [12]. It acts by increasing glutamatergic transmission [13], synaptogenesis [14], synaptic plasticity [15], and neurotrophic factor expression [16] in key brain regions mediating mood regulation, such as the prefrontal cortex (PFC) and the hippocampus. However, its side effects have limited its clinical application [17].

Adiponectin is an adipocyte-secreted hormone that can cross the blood-brain barrier. It can act on its specific receptors in the hypothalamus to increase food intake and decrease energy metabolism [18]. Besides, adiponectin receptors are also present in several brain regions, including the hippocampus and the medial PFC [19]. Notably, intracerebral (i.c.v.) recombinant adiponectin infusion promotes dendritic arborization, spinogenesis, and neurogenesis in the hippocampal dentate gyrus (DG) [20], modulates hippocampal synaptic plasticity [21, 22], and elicits antidepressant response in normal mice [19]. Remarkably, it was recently demonstrated that the systemic administration of AdipoRon, a selective agonist of adiponectin receptors, can elicit an antidepressant response in depressed mice [23]. AdipoRon can bypass the blood-brain barrier [24], indicating its direct effect on the brain. The modulation of the adiponectin signaling pathways, therefore, has unmasked a novel antidepressant strategy.

In this review, we summarize the pieces of evidence showing the effects of the adiponergic system on modulating neuroplasticity in the central nervous system.

## Adiponectin

**Fig. 1:**
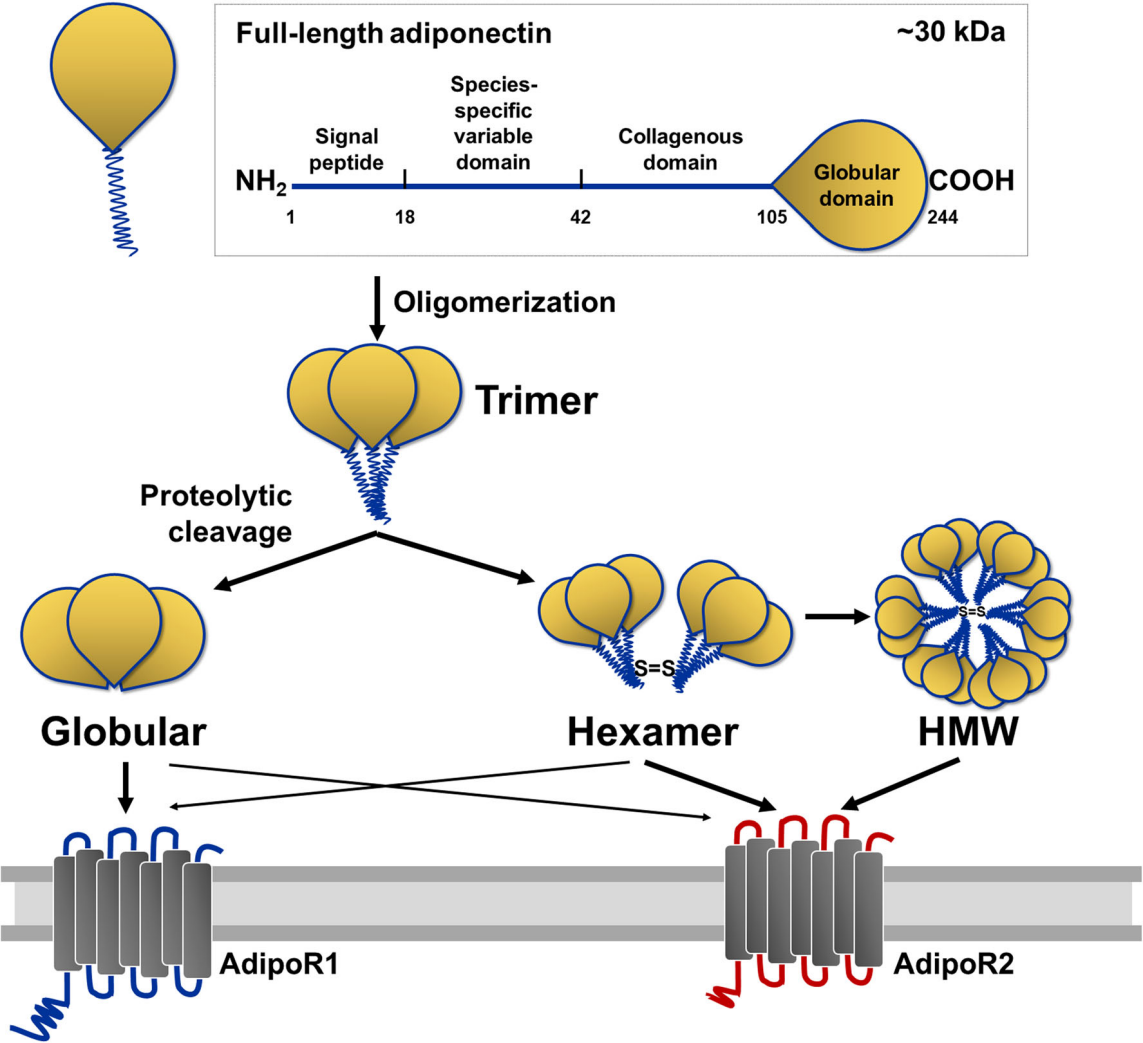
Adiponectin structure and its receptors. A full-length adiponectin (~ 30 kDa) consists of a globular domain, a collagenous domain, a species-specific domain, and a signal peptide. Oligomerization facilitates the formation of the trimer, hexamer, and high-molecular weight (HMW) adiponectin. Full-length adiponectin can undergo proteolytic cleavage, whose proteolytic fragment corresponds to the globular adiponectin. AdipoR1 has a greater affinity for the globular form, whereas AdipoR2 has a moderate affinity for both globular and full-length forms

Adiponectin is the most abundant plasma protein in the circulation. It is released by mature white adipocytes and takes up about 0.01% of the total plasma proteins in human [25]. Adiponectin circulates as a trimer, hexamer, and high-molecular weight multimers, which are the major forms in mammals **(**Fig. 1**)** [26, 27]. Still, only trimer and hexamer are permeable to the blood-brain barrier and their concentrations in the cerebrospinal fluid compared to serum levels are approximately 1- to 4000-fold [18]. There are two adiponectin-specific receptors identified: adiponectin receptor 1 (AdipoR1) and receptor 2 (AdipoR2) [28]. AdipoR1 and AdipoR2 have differential affinities for different adiponectin oligomeric forms. AdipoR1 has a greater affinity for the globular form, whereas AdipoR2 has a moderate affinity for both globular and full-length forms [28]. AdipoR1 is highly expressed in brain structures like the hippocampus, the PFC, the amygdala, the hypothalamus, and the ventral tegmental area [18, 19, 29], whereas AdipoR2 is more restricted to regions such as the hippocampal DG [30] and hypothalamus [18].

Adiponectin acts on the liver, the muscle, the heart, the adipocyte, and the blood vessel with antidiabetic, anti-inflammatory, antiatherogenic, and cardiovascular protective properties [31–34]. In the brain, adiponectin promotes food intake through the activation of its receptors in the hypothalamus [18]. Paradoxically, increased adipocyte size does not increase adiponectin secretion in obesity [35, 36], mainly due to hypoxia, cellular inflammation, and nutrient deprivation in the oversized adipocytes. The increase in pro-inflammatory cytokines suppresses adiponectin synthesis, leading to hypoadiponectinemia. Noticeably, hypoadiponectinemia is observed in type 2 diabetes mellitus (T2DM) [37], in which the adiponectin level also correlates with the comorbid depressive symptom severity [38]. Metabolic and mood disorders are, therefore, intertwined by the adiponergic system.

## Changes in Peripheral Adiponectin Levels in Patients with Depression

An association among peripheral adiponectin levels and MDD has been suggested in different populations and health conditions (Table 1). A study consisting of cross-sectional (*n* = 575) and longitudinal (*n* = 262) analyses has shown that current episode of MDD, symptom severity, and history of depression in middle-aged women were all linked to the low adiponectin levels over a 5-year follow-up [39]. In depressed women, the adiponectin levels were sharply reduced by 25% and remained low over a 24-h period when measured hourly [40]. The correlations between low adiponectin levels and depression severity were also shown in men [41] and elderly patients [42]. This adiponectin-depression relationship was briefly summarized by a recent meta-analysis, illustrating a significant decrease in the adiponectin levels in depressed patients compared to controls, in both males and females [49].

**Table 1:** Changes in peripheral adiponectin levels in patients with depression

Authors (year) (ref)	Study design	Population		Associations	
		Sex (*n*)^a^	Associated condition	Depression indices	Adiponectin levels
Everson-Rose et al. (2018) [39]	Cross-sectional	♀ (575)	–	Current depression Symptom severity History of depression	↓
Cizza et al. (2010) [40]	Case-control	♀ (23)	–	History of depression Cumulative duration of depression	↓
Leo et al. (2006) [41]	Case-control	♀ ♂ (32)^b^	–	Current depression Symptom severity	↓
Diniz et al. (2012) [42]	Case-control	♀ (37) ♂ (10)	Elderly subjects	Current depression Symptom severity	↓
Laake et al. (2014) [43]	Cross-sectional	♀ (793)^c^ ♂ (976)	T2DM	Current depression	↓ (trend *p =* 0.09)
Herder et al. (2017) [44]	Cross-sectional	♀ (55) ♂ (84)	T1DM	Symptom severity	No association
		♀ (97) ♂ (198)	T2DM	Symptom severity	↓
Herder et al. (2018) [38]	Cross-sectional	♀ (227) ♂ (162)	T1DM	Symptom severity	No association
		♀ (88) ♂ (116)	T2DM	Symptom severity	↓
Yang et al. (2018) [45]	Cohort	♀ (117) ♂ (138)	Ischemic stroke	Poststroke depression at 3 months	↓ at baseline
Fábregas et al. (2016) [46]	Cohort	♀ (26) ♂ (24)	Hepatitis C	MDD at 3 months	↓ at baseline
Tunçel et al. (2016) [47]	Case-control	♀ (23) ♂ (7)	Adolescents (11–18 y.o.)	Current depression	No association
Rebelo et al. (2016) [48]	Cohort	♀ (177)	Pregnant women	Perinatal depression	No association

^a^Total *n* number for cross-sectional and cohort studies, *n* number of cases for case-control design

^b^Individual numbers of males and females are not reported

^c^From the total sample of 1769 subjects (male and female), 1227 were included in the analysis

Adiponectin is also known as an anti-inflammatory cytokine. Metabolic disorders and cardiovascular diseases are marked by altered adiponectin levels [50, 51]. Coincidentally, depression is often comorbid with these disease states [52]. A large cross-sectional study (*n* = 1227) reported a correlating trend (*p* = 0.09) of the reduced adiponectin levels in early-stage T2DM and the severity of depression [43]. This association was later confirmed by two other studies, in which both high molecular weight to total adiponectin ratio [44] and total adiponectin concentrations [38] were correlated with the severity of depression in T2DM, but not in T1DM. In ischemic stroke patients, lower adiponectin levels at admission increased three times the risk of developing post-stroke depression [45]. In hepatitis C patients, higher adiponectin levels were associated with a lower incidence of MDD [46].

Interestingly, in consonance with the heterogeneity of depressive disorders, peripheral adiponectin levels might not be a ubiquitous biomarker for all depressive states. Depressed patients at the adolescent age ranging from 11 to 18 years old displayed comparable adiponectin levels to healthy age-matched controls [47]. Additionally, adiponectin levels increased along with the pregnancy and the postpartum period, but with no correlation with the incidence of depressive symptoms [48]. This idea is also illustrated in rodent studies using different depressed animal models. Mice subjected to chronic unpredictable mild stress [53] or chronic corticosterone administration in drinking water [23, 54] did not reduce peripheral levels of adiponectin. However, the depressed mouse model induced by chronic social defeat stress had a signficant reduction in peripheral adiponectin levels, which was inversely correlated to the increased severity of depressive behavior [19, 55]. These differences in rodent studies are likely due to the variations in the paradigms used for inducing depressive-like behaviors.

In summary, the evidence so far has suggested that decreased peripheral adiponectin levels can potentially be linked to major depressive disorder and depression co-morbid with some metabolic and cardiovascular disorders.

## Central and Peripheral Modulations of the Adiponergic Pathway on Antidepressant Effects

The effects of antidepressant treatments over peripheral adiponectin levels are controversial in clinical studies. A short treatment period of 4 to 5 weeks by several classes of antidepressant drugs did not largely affect [47, 56, 57], but with chances of reducing [58], adiponectin levels in depressive patients. On the other hand, MDD-remitted patients who had undergone selective serotonin reuptake inhibitor (SSRI) or serotonin-norepinephrine reuptake inhibitor (SNRI) treatments for at least 6 months showed increased levels of adiponectin and decreased levels of tumor necrosis factor alpha (TNF-α) when compared to healthy, matched controls [59]. Nonetheless, the improvements in depressive symptoms after long-term non-pharmacological, behavioral-cognitive therapy for T1DM and T2DM with comorbid depression and distress were not associated with increased adiponectin levels in the 12-month follow-up [60]. The fact that adiponectin is the most abundant plasma protein may hinder the detection of subtle changes in adiponectin levels, leaving only substantial alteration in the peripheral adiponectin levels as statistically detectable.

From another perspective, manipulation of peripheral adiponectin levels appears to elicit antidepressant effects in rodents. Rosiglitazone is a known effective antidiabetic agent, selectively agonizing the peroxisome proliferator-activated receptor gamma (PPARγ), an upstream positive regulator of adiponectin [61]. Rosiglitazone cannot bypass the blood-brain barrier. Systemic administration of rosiglitazone resulted in adiponectin-dependent antidepressant response in mice [55]. Moreover, systemic administrations of rosiglitazone within 24 h significantly increased peripheral adiponectin levels, which was necessary and sufficient to elicit an antidepressant response [55].

Besides, rodent studies have not only demonstrated the necessity of adiponectin in exercise- and environment-induced antidepressant effects [54, 62, 63] but also hint on the fact that increased adiponectin level in the central nervous system is associated with the antidepressant response. Particularly, voluntary wheel running for 14 days induced antidepressant effects in wild-type mice with increased adiponectin concentrations in the hippocampal DG, but not in the serum [60]. Similarly, environmental enrichment prevented anxiety and depression-like states in chronically-stressed mice with a four-fold increase in the cerebrospinal adiponectin levels, whereas plasma adiponectin levels remained unchanged [54]. These animal studies have shed light on the possible roles of the adiponergic system in inducing antidepressant effects [64, 65].

Importantly, direct activation of the central adiponergic pathway shows antidepressant effects. Central activation of the central adiponergic pathway by overexpressing adiponectin [62] or i.c.v. infusion of recombinant adiponectin consistently elicited antidepressant responses [19, 54, 63]. Strikingly, these animal studies have also shown that the adiponectin-induced antidepressant response was observable within hours.

AdipoRon is an orally active molecule that selectively agonizes the AdipoR1 and AdipoR2 [24]. As adiponectin, it exerts antidiabetic [24], anti-inflammatory [66], and cardiovascular protective properties [67]. AdipoRon can also bypass the blood-brain barrier [23] and act on brain regions like the hippocampus [30] and the ventral tegmental area [29]. Congruently, chronic administration of AdipoRon promoted hippocampal adult neurogenesis and results in antidepressant response in several animal models of depression [23]. Altogether, this data indicate that targeting adiponectin receptors and activating the adiponergic pathway are potential strategies for developing antidepressant drugs.

## Potential Mechanisms of the Antidepressant Effects of Adiponectin

### Effects of Adiponectin on Neurogenesis

In the adult mammalian brain, the sub-granular zone of the hippocampal DG contains a reservoir of neural stem cells. Granule neurons are continuously generated from these progenitors via adult hippocampal neurogenesis [68–73], which can integrate into the existing neural circuit [74–77]. Conventionally, depression is closely related to brain structure integrity [78], increased cellular stress [10], and increased dendritic and spine atrophy [10]. It was further postulated that adult hippocampal neurogenesis could antagonize stress and depression [79]; concurrently, antidepressant drugs are effective in promoting adult hippocampal neurogenesis [80–83]. Given so, the endeavor to reveal the role of adiponectin in structural plasticity were made thereafter.

Current opinion towards adiponectin is far more than an adipocyte-secreted endocrine hormone, but a neurotrophic factor, such that disruption of the adiponectin signaling pathway in the hippocampus impairs neurogenesis and cognitive functions [20, 62, 84]. The neurotrophic effect of adiponectin was first demonstrated in adult hippocampal stem cells, which expressed both AdipoR1 and AdipoR2 [85]. Adiponectin promoted proliferation, but not differentiation nor survival, *in vitro* via the p38 mitogen-activated protein kinases (MAPK)/glycogen synthase kinase (GSK)-3β/β-catenin signaling pathway [85]. An adiponectin null mutant had reduced cell proliferation, differentiation, and survival in the hippocampus [20], whereas infusing adiponectin [20] or overexpressing adiponectin [62] in the mouse brain could promote cell proliferation in the hippocampal DG.

Physical exercise promotes adult neurogenesis in the hippocampus [86, 87]. It induces the release of neurotrophic factors such as the brain-derived neurotrophic factor (BDNF) [88, 89] and the insulin-like growth factor-1 (IGF-1) [90]. Rodents perform better in spatial recognition [91, 92] and have better executive functions [93] after exercise. In the study dissecting the role of adiponectin in exercise-induced antidepressant effect, the exercise-induced adult hippocampal neurogenesis was abolished in adiponectin-deficient mice [62]. The role of adiponectin as a mediator in exercise-promoted adult hippocampal neurogenesis is re-confirmed using streptozotocin to induce diabetes in adiponectin-deficient mice. Exercise could restore impaired hippocampal neurogenesis in wild-type diabetic mice, but not in adiponectin-deficient diabetic mice [84]. The neurogenic effects are possibly mediated by activating the AdipoR1/APPL1/AMPK pathway as shown by Yau and colleagues [62].

### Effects of Adiponectin on Dendritic Complexity and Spinogenesis

Synaptic connections between neurons are predominantly tied up by dendritic spines. Spinogenesis is precisely regulated in response to stress, which consequently promotes rewiring of the neural network [94]. Depression is associated with dendritic spine pathology in the hippocampus [95–97]. Spinogenesis is often dysregulated in chronically stressed animals [98, 99]. Antidepressants can reverse spine and dendrite atrophy in animal models of depression [100, 101], leading to the idea that dendritic and spine atrophy could contribute to symptoms of depression [9, 102, 103]. Therefore, unraveling the role of adiponectin in spinogenesis can shed light on depression.

In addition to the data above, adiponectin promotes dendritic growth, arborization, and spine remodeling in the hippocampal DG [20]. Adiponectin null mutants had a reduced dendritic length, branching, and spine density of granule neurons, particularly in granule neurons generated during embryonic development [20], whereas i.c.v. infusion of adiponectin for a week promoted spinogenesis and dendritic complexity in adult-born granule neurons [20]. Moreover, upregulating AdipoR1/Nogo-66 receptor 1 (NgR1) signaling pathway by an adiponectin homolog, osmotin, could also enhance neurite outgrowth and synaptic complexity in the hippocampus in an Alzheimer’s disease mouse model [104].

Adult hippocampal neurogenesis is impaired by stress and depression, whereas multiple rodent studies have demonstrated the neurogenic and antidepressant effects of adiponectin. The accumulated evidence has suggested that enhanced structural plasticity may be a critical factor in the adiponectin antidepressant properties.

### Effects of Adiponectin on Synaptic Plasticity

Altered synaptic integrity underlies the structural changes, specifically reduced white matter integrity [78] and the mean hippocampal volume [105], reported in MDD patients. MDD patients have fewer spines in the PFC as well as reduced expression of genes participating in synaptic plasticity [106]. Such disturbance in synaptic integrity could deter synaptic transmission. Long-term potentiation (LTP) and long-term depression (LTD) are standard evaluations of synaptic plasticity [107]. Chronic stress, a conventionally accepted risk factor for depression [108], impairs hippocampal LTP [109, 110] and facilitates LTD [111] in various stress-induced depressed rodent models.

Conversely, chronic treatment with standard antidepressants prevents stress-induced hippocampal LTD [111] and stress-induced disturbances in synaptic proteins, such as PSD-95 and synapsin I [112]. Considerably, a single dose of ketamine induces fast antidepressant response and restores the LTP and NMDAR-dependent excitatory postsynaptic current in depressed mice [110]. Altogether, it indicates that altered synaptic plasticity plays a significant role in the depression pathophysiology and, concurrently, represent a potential target for rapid-acting antidepressants.

The effect of adiponectin on modulating synaptic plasticity is summarized in Table 2. At present, bidirectional effects of activating the adiponectin receptors on synpatic plasticity have been found. i.c.v. adiponectin infusion increased LTP and prevented LTD in the DG [21]. However, incubation of acute hippocampal slices with AdipoRon further dampened LTP in the *Cornu Ammonis* 1 (CA1) [22]. Factors affecting the adiponectin receptor-mediated synaptic transmission are not completely understood. The differential expressions of AdipoR1 and AdipoR2 across several brain structures [19] may indicate that they play different roles in synaptic transmission. AdipoRon could increase extinction learning with a decrease in DG neuron intrinsic excitability through an AdipoR2-dependent mechanism [30]. Congruently, ventral tegmental area (VTA) infusion of AdipoRon prevented stress-induced anxiety-like behavior with a reduction in dopaminergic neuron activity, which was mediated by AdipoR1-dependent activation [29]. Further investigations on the mechanisms of actions will ultimately demonstrate the adiponectin signaling pathway modulating synaptic plasticity in the brain.

**Table 2:** Effects of adiponectin on synaptic plasticity

Authors (year) (ref)	Subjects (age)	Methods	Site	Electrophysiological findings	Behavioral outcomes
Weisz et al. (2017) [22]	Adult and young mice (C57BL/6J)	Extracellular recording and whole-cell patch clamping	CA1	↓ Paired-pulse ratio ↓ Long-term potentiation ↓ AMPA/NMDA ratio (only in adult mice)	N/A
Sun et al. (2018) [29]	Adult mice (C57BL/6J)	*In vivo* single-unit electrophysiological extracellular recording	VTA	↓ Population activity^a^ ↓ Average spontaneous firing rate	Reduced the expression of anxiety-like behaviors
Zhang et al. (2017) [30]	Adult mice (C57BL/6J)	Whole-cell patch clamping	DG	↓ Number of action potential ↑ Rheobase current ↑ Negative resting membrane potential	Improved contextual fear memory extinction
Pousti et al. (2018) [21]	Adult rats (Wistar)	*In vivo* extracellular recording	DG	↑ Long-term potentiation ↑ Paired-pulse ratio ↑ Baseline Prevented long-term depression	N/A

^a^The number of spontaneously active neurons recorded per track

## Conclusion and Perspectives

**Fig. 2:**
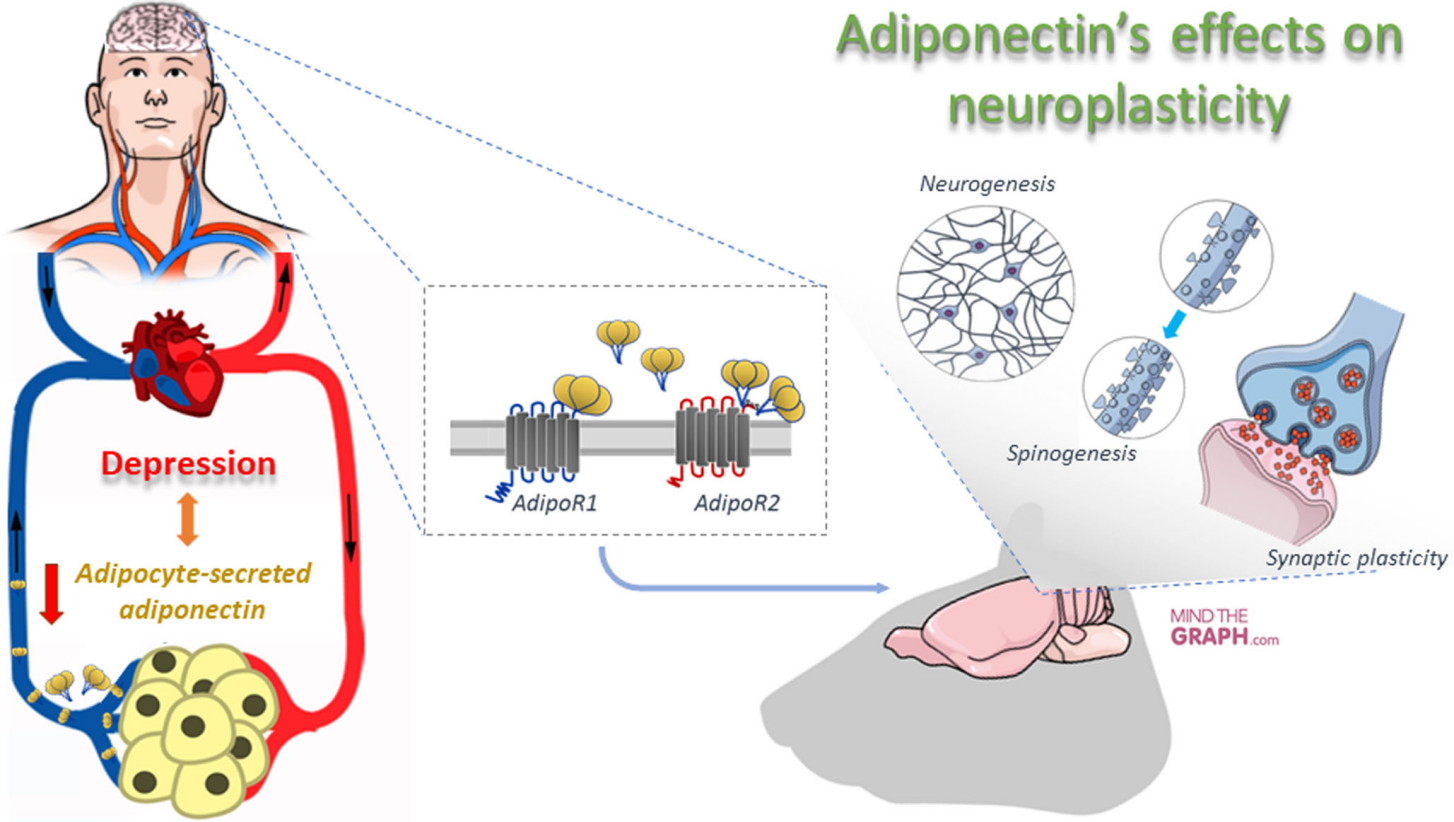
Proposed beneficial effects of adiponectin in depression. A large body of clinical research have been implicating low peripheral adiponectin levels with depression, and so antidepressant effects of adiponectin may be linked to its effect on promoting neuroplasticity.

Antidepressant effects of adiponectin have been shown in depressed rodent models. So far, it has been reported that adiponectin mediates physical exercise and enriched environment-induced antidepressant response, likely due to its promoting effects on adult hippocampus neurogenesis or neurotrophic properties. Animal studies have demonstrated a region-specific effect of AdipoR1 and AdipoR2 on anxiety-like behaviour and fear memory extinction, respectively. The findings of AdipoR1/2-dependent modulation of synaptic plasticity and neuronal excitability have suggested differential roles of AdipoR1 and AdipoR2 in the brain. So far, accumulating evidence has suggested that changes in functional neuroplasticity following adiponectin signaling activation could also underly its antidepressant effects as reported from the current literature (Fig. 2).

This is in consonance with the current systemic conceptualization of depression in terms of its neuroplasticity changes [8–10] that, when counteracted, may result in sustained antidepressant responses. Nevertheless, adiponectin is also a metabolic regulator with insulin-sensitizing, anti-inflammatory, and cardioprotective properties, bridging the correlation between depression and metabolic disorders. Hence, it is tempting to think that targeting the adiponergic system may not only induce a rapid and sustained antidepressant effect but also regulate the metabolic dysfunction commonly associated with depression.

Even though experimental studies have just started unraveling the adiponectin mechanisms of action in neuroplasticity, and some antagonisms remain to be explained, the adiponergic system stands as a promising antidepressant target with fast response, small side effects, and capability of improving the comorbid metabolic syndromes.

## Electronic Supplementary Material

Supplementary Table 2 (extended) (DOCX 91 kb)
