# Supplementary material for: Increasing Adiponergic System Activity as a Potential Treatment for Depressive Disorders
Source: Mol Neurobiol. 2019 May 28;56(12):7966–76. doi: 10.1007/s12035-019-01644-3 (PMC6834732; doi:10.1007/s12035-019-01644-3)
Supplement: Supplementary file 1 — (DOCX 91 kb) [file 12035_2019_1644_MOESM1_ESM.docx]

Table 1. Effects of adiponectin on synaptic plasticity

| **Authors (year)** | **Subjects**  **(age)** | **Technique** | **Protocol/**  **Measures** | **Site** | **Drugs (concentration)** | **Treatment** | **Behavior** | **Findings** | |
| --- | --- | --- | --- | --- | --- | --- | --- | --- | --- |
|  | **Mice**  **(C57BL/6J)** |  |  |  |  |  |  |  |  |
| Weisz et al. (2017) | Wild type:  Young (2-4 w)  Adult (5-8 w) | Extracellular recording  Patch Clamp | LTP, Paired-pulse ratio (PPR), Input-output curve (IO)  AMPA/NMDA ratio | CA1 | AdipoRon (1.5, 3, 30 µM) | 2-h incubation^a^ | N/A | AdipoRon modulates synaptic plasticity both through pre-synaptic (PPR) and post-synaptic mechanisms (LTP) in the CA1 region of young and adult mice | |
|  |  |  |  |  |  |  |  | **Young mice**  PPR (30 µM)  LTP (30 µM)  AMPA/NMDA not reported | **Adult mice**  PPR (30 µM)  LTP (3 and 30 µM)  AMPA/NMDA ratio (30 µM) |
| Zhang et al. (2017) | Adult (8-10 w):  Wild type  *Adipo^- / -^*  *AdipoR2^- / -^*  *AdipoR1^flox/flox^* | Patch Clamp | Number of action potential (AP)  Rheobase current  Input resistance  Resting membrane potential (RMP) | DG | Adiponectin (0.25 µg)  AdipoRon (15 µM) | Intra-DG 30 min before the behavior  Bath perfusion | Contextual fear conditioning and extinction | Adiponectin decreases intrinsic excitability of DG granule neurons through activation of AdipoR2, modulating contextual fear memory extinction | |
|  |  |  |  |  |  |  |  | ***Adipo^- / -^* Vs Wild-type**  Number of AP  Input resistance  Rheobase current  Extinction  **Wild-type + AdipoRon**  **Vs Vehicle**  Number of AP  Rheobase current  Negative RMP  ***AdipoR1^flox/flox^* with intra-DG AAV-Cre-GFP**  No detectable differences in behavior compared to *AdipoR1^flox/flox^* with intra-DG AAV-GFP  ***AdipoR2^- / -^* Vs Wild type**  Fear expression  Extinction  ***AdipoR2^- / -^* Vs Wild-type**  Number of AP  Input resistance  Rheobase current | ***Adipo^- / -^* + Adiponectin Vs Vehicle**  N/A  N/A  N/A  Extinction  ***Adipo^- / -^* + AdipoRon**  **Vs Vehicle**  Number of AP  Rheobase current  Negative RMP  ***AdipoR2^- / -^* + Adiponectin Vs Vehicle**  Fear expression  Extinction  ***AdipoR2^- / -^* + AdipoRon Vs Vehicle**  No detectable differences in intrinsic excitability parameters |
| Sun et al. (2018) | Adult (8-12 w):  Wild type  *Adipo^+/-^*  *AdipoR1^flox/flox^/ DAT^IREScre^* | *In vivo* single-unit electrophysiological extracellular recording | Population activity^b^  Average spontaneous firing hate (Hz)  Average percentage burst firing^c^ | VTA | Adiponectin:  - 0.15 µg for ephys  - 0.1, 0.3 µg for behavior  AdipoRon (0.1 or 1.0 nM) | Intra-VTA infusion 30 min before behavior or ephys recording | Acute restraint stress  Elevated plus maze  Light-Dark box | Adiponectin acts on VTA dopamine neurons, modulating neuronal activity and anxiety-like behaviors through activation of AdipoR1 receptors | |
|  |  |  |  |  |  |  |  | **Adiponectin in wild-type non-stressed mice**  Population activity  Average firing rate  Open-arm entries  Light-compartment time  **Acute restraint stress in wild-type mice**  Population activity  Open-arm time  Latency to the light compartment  ***Adipo^+/-^***  Open-arm time  Open-arm entries  Latency to the light compartment  Firing rate | **AdipoRon in wild-type non-stressed mice**  Population activity  Average firing rate  **AdipoRon in wild-type stressed mice**  Population activity  Open-arm time  Latency to the light compartment  Open-arm entries  Light-compartment time  ***AdipoR1^flox/flox^/ DAT^IREScre^* in stressed mice**  Open-arm time  Open-arm entries  Latency to the light compartment  Firing rate |
|  |  |  |  |  |  |  |  | **Adiponectin in *AdipoR1^flox/flox^/ DAT^IREScre^* in non-stressed mice** | |
|  |  |  |  |  |  |  |  | No detectable differences in neuronal activity nor in anxiety-like behaviors compared with controls | |
|  | **Rats**  **(Wistar)** |  |  |  |  |  |  |  | |
| Pousti et al. (2018) | Adult | *In vivo* extracellular recording | LTP, Paired-pulse ratio (PPR), LTD | DG | Adiponectin (600 nM) | I.c.v. |  | I.c.v. infusion of adiponectin increases synaptic plasticity in the hippocampal DG | |
|  |  |  |  |  |  |  |  | LTP  PPR  Baseline | Prevent LTD |

^a^ A 15-min perfusion time was also tested, without significant impact over basal synaptic transmission and HFS-induced LTP.

^b^ The number of spontaneously active dopamine neurons recorded per track.

^c^ Defined as the occurrence of two consecutive spikes with an interspike interval <80 ms
